# The first-generation ABSORB BVS: awaiting dissolving outcomes

**DOI:** 10.1007/s12471-017-1042-8

**Published:** 2017-09-14

**Authors:** J. Elias, I. M. van Dongen, J. P. S. Henriques

**Affiliations:** Academic Medical Center, University of Amsterdam, Amsterdam, The Netherlands

We thank Dr Shah for his response to our meta-analysis. As Dr Shah points out, in our meta-analysis the ABSORB II trial was the only study that reported 3‑year follow-up. All the other trials had a follow-up duration of 2 years or less, and the AIDA trial reported a median follow-up of 2 years [1]. Therefore, the very-long-term clinical efficacy and safety of the bioresorbable vascular scaffold (BVS) are currently unknown.

Although the low numbers of events between 2–3 years of follow-up in both the ABSORB-Japan and ABSORB-China trials might be reassuring [2, 3], in the ABSORB II trial there were four scaffold thromboses in the Absorb BVS arm (*n* = 313) between 2–3 year follow-up (rate of ~1.2%) [4]. Furthermore, several limitations of the ABSORB-Japan and ABSORB-China trials must be pointed out. Both trials only included about 15% (*n* = 875) of the patients analysed in our meta-analysis, resulting in a weight of less than 5%. Furthermore both trials included highly selected patients and lesions and the results are therefore not applicable to daily clinical practice.

Also, in the ABSORB-Japan trial almost 42% of the BVS patients were still on dual antiplatelet therapy (DAPT) at 3‑year follow-up. In addition, no statement was given on the reasons for DAPT prolongation and whether patients who discontinued DAPT had an increased risk for stent thrombosis. In the ABSORB-China trial about 20% of patients were still on DAPT at 3‑year follow-up. Although it is currently unknown if prolongation of DAPT will be able to prevent (late) scaffold thrombosis, variance in DAPT duration limits the results of the trials.

Nonetheless, even if the device thrombosis rate of the Absorb BVS is low between 2–3 years of follow-up, the cumulative device thrombosis rate for the Absorb BVS remains high compared with the Xience metallic stent, and any potential benefit is still unnoticed. A decline in scaffold thrombosis is probably something to be expected, since less of the scaffold is present during follow-up leading to fewer events. However, the real questions are what will happen in the following years after the scaffold is completely dissolved and whether the benefits will outbalance the (early) risks.

We agree with the author that further (longer and more) follow-up should be awaited to fully assess the possible benefit of the dissolving BVS. However, the reduction in scaffold thrombosis rate between 2–3 year follow-up is at best encouraging to further extend technical improvement and provide newer insights to improve outcomes. Still, until more long-term data are presented, with current knowledge the use of the Absorb BVS should be restricted to regulated clinical studies, if to be used at all.
